# Tunable Structure and Properties of Segmented Thermoplastic Polyurethanes as a Function of Flexible Segment

**DOI:** 10.3390/polym11121910

**Published:** 2019-11-20

**Authors:** Manuel Asensio, Victor Costa, Andrés Nohales, Otávio Bianchi, Clara M Gómez

**Affiliations:** 1 Institute of Materials Science, University of Valencia, 46980 Paterna, Valencia, Spain; manuel.asensio@uv.es; 2 R&D Department UBE CORPORATION EUROPE, S.A., 12100 Castellon, Spain; v.costa@ube.com (V.C.); a.nohales@ube.com (A.N.); 3 Chemical Engineering Department, University of Caxias do Sul, Caxias do Sul 95070560, Brazil; otavio.bianchi@gmail.com

**Keywords:** thermoplastic polyurethane, flexible segment, degradation, phase segregation, mechanical and thermal properties

## Abstract

Segmented thermoplastic polyurethanes (PUs) were synthetized using macrodiols with different functional groups (carbonate, ester, and /or ether) as a segment with a molar mass of 1000 and 2000 g/mol, and 4,4’-diphenylmethane diisocyanate (MDI) and 1,4-butanediol as a rigid segment. The polyurethanes obtained reveal a wide variation of microphase separation degree that is correlated with mechanical properties and retention of tensile properties under degradation by heat, oil, weather, and water. Different techniques such as differential scanning calorimetry (DSC), dynamic mechanical analysis (DMA), Fourier transform infrared (FTIR), and synchrotron small-angle X-ray scattering (SAXS) were used to determine rigid-flexible segments’ phase behaviour. Retention of tensile properties determines the stability of the samples under different external factors. This work reveals that pure polycarbonate-based macrodiols induce the highest degree of phase miscibility, better tensile properties, hardness shore A, and retention of tensile properties under external agents.

## 1. Introduction

Polymer materials classified as polyurethanes are one of the main synthetic materials employed nowadays. They are characterized by having a high proportion of urea and/or urethane linkages in their structure. Depending upon the components, composition, and synthesis procedure employed to obtain them, a great range of material properties is attained. They are used in a wide range of applications as they can be produced as synthetic rubbers, adhesives, foams, fibers, protective coating, elastomers, biomaterials, semi-permeable membranes, rigid devices, and sealants [1,2,3,4,5,6].

In particular, segmented polyurethanes elastomers (PUs) are block copolymers formed with alternating flexible and rigid segments giving a two phase separated structure that is responsible for the final properties. The flexible segment is a macrodiol of low glass transition temperature such as polyether, polyester, or polycarbonate. The rigid segment is formed by a diisocyanate and a low-molar mass diol that acts as a chain extender. The flexible segment imparts elasticity and flexibility at room temperature, while the rigid segment displays hydrogen bonding interactions, thus forming a physically cross-linked network contributing to mechanical reinforcement. The elastomeric behavior of these materials is closely related to microphase separation of rigid-flexible segments. A detailed study to understand structure–properties correlations is crucial to determine the applications and end-use of these materials and to design new structures. Thus, the final properties are tightly related to the type and composition of raw materials employed and the composition of the soft and hard phases, and can be widely tunable for the application to be used. Moreover, PUs can be obtained by different production procedures (with or without solvent, casting, injection, reactive extrusion, spraying) in order to fabricate objects of varying sizes and shapes. Temperature increase causes disruption of the hydrogen bonding and permits melt processing. Another great advantage is that they can be easily recycled [1,2,3,4,5,6,7].

The components, composition, and production procedure of polyurethane will be the key to obtain a material for each specific application. Changing the raw materials and relative proportions, that is, flexible segment or macrodiol, dicyanate, chain extender, and/or proportion of rigid-to-flexible segment; synthetic methods; and reaction conditions, allows us to modulate polyurethane properties, especially tensile strength, elongation at break, hardness, and extension of chemical or physical degradation. Focusing on the composition of the flexible segment, polyether or polyester macrodiols are commonly used as flexible segments owing to the low price and easy handling, as they are available as liquids. Polyetherdiol flexible segment imparts high resistance to hydrolysis, but gives low mechanical strength in contrast with polyester polyol-based PUs [8,9,10,11]. Polycarbonate flexible segment is more thermal stable than the polyether one, showing only minimal chemical or physical degradation and high heat and mechanical resistance [12]; however, it is hygroscopic and the water absorbed disrupts the hydrogen bonding in the ordered PU domains with a plasticizing effect [13,14].

In general, polyurethanes are used in many different applications as the formulation developed shows outstanding properties such as high solvent and mechanical resistance (hardness/flexibility compromise), excellent adhesion onto various substrates, fast film formation, and excellent weathering resistance. Studies on degradation/stability have been mainly centered on biomedical applications [4,6,12,13,14]. These properties are tightly correlated with the biphasic nature of segmented polyurethanes in the hard and soft phase. This, in turn, depends upon the chemical nature and composition of both phases. Flexible segment polyether and polyester based PUs are susceptible to degradation under hydrolytic and oxidative environments. Degradation of polyurethanes caused by different factors such as weather, water, oil, or heat may lead to a chaotic dysfunction of these materials owing to changes in the polymer structure [8,9,10,11,12,13,14,15,16,17]. Investigation on degradation, morphology, thermal, and mechanical behavior is crucial to determine the end-use of these materials. 

The objective of this paper is to explore the possibility to synthetize segmented polyurethane elastomers changing properties as a function of flexible segment. Thus, it is possible to obtain PU with tunable properties depending on the desired application. The particular interest was to investigate the effect of different molecular structures and molar masses of flexible segments or macrodiols on the degree of phase separation between hard and flexible segments in PU. So, we synthetized different segmented polyurethanes with 4,4’-diphenylmethane diisocyanate (MDI) as the rigid segment and butanediol as the chain extender. The flexible segment is based on three different combinations of functionalities: carbonatediol, ester, and ether. Thus, five different flexible segments based on polycarbonatediol (PCD), polyetherdiol, and polyesterdiol functionalities with two different molar masses were used to synthesize the PU under investigation. A deep study correlating microstructure-phase separation-properties was carried out using different techniques such as differential scanning calorimetry (DSC), dynamic mechanical analysis (DMA), Fourier transform infrared (FTIR), and synchrotron small-angle X-ray scattering (SAXS). Moreover, studies on resistance to degradation in different media and external factors such as heat, weather, oil, and water were assessed by determining the tensile properties’ retention.

## 2. Materials and Methods 

### 2.1. Materials

In this study, we prepared different segmented polyurethanes by changing the molar structure and the molar mass of the flexible segment. The rigid segment consists of 4,4’-diphenylmethane diisocyanate (MDI) and 1,4-butanediol (BD) as the chain extender, which were obtained from Sigma Aldrich (Barcelona, Spain). 

The flexible segments or macrodiols with an average molar mass of 1000 and 2000, respectively, are as follows: poly(hexamethylene) carbonate diol Eternacoll® UH (UH100 and UH200), poly(hexamethylene–pentamethylene) carbonate diol Eternacoll® PH (PH100 and PH200), and poly(hexamethylene–caprolactone) carbonate diol Eternacoll® UHC (UHC100 and UHC200) supplied by UBE Chemical Europe (Castellón, Spain); poly(tetramethylene ether) glycol (PTMG100 and PTMG200) supplied by Sigma Aldrich; and polycaprolactone polyester diol Capa ™ 2100 (PCL100) and Capa ™ 2200 (PCL200) supplied by Perstorp Holding AB (Malmö, Sweden).

The main characteristics such as molecular structure; molecular weight; and melting and glass transition temperatures, *T*_m_ and *T*_g_, respectively, of the pure materials are summarized in Table 1. 

All materials were used as received and kept in a dry box to avoid humidity.

### 2.2. Polyurethane Synthesis

Thermoplastic polyurethanes were obtained via a two-step, prepolymer synthesis method [16]. In the first step, the macrodiol and an excess of diisocyanate were poured in a reactor at a temperature of 70 °C over 1 h in an argon atmosphere to form a prepolymer of polyol endcapped with diisocyanate groups. In a second step, butanodiol at a molar ratio of NCO/OH = 1.03 was added to the prepolymer in a SpeedMixerTM Dac 600.1 FVZ mixer (Landrum, SC, USA) at room temperature for 1 min at 2250 r.p.m. The subsequent PU solution was cast on aluminium moulds at 90 °C, and was compression moulded at a pressure of 50 bars and temperature of 100 °C for 24 h using a water-cooled hydraulic Carver press model 4128CE S/N 4128-220 (Wabash, IN, USA). The cooling procedure was kept uniform by carefully controlling the water flow rate. PU plaques were 2 mm thick. 

A molar ratio of 1:3:2 (polyol/MDI/BD) was used for the synthesis of all polyurethanes. Samples were tested in the as-moulded condition only. The experimental results are the mean value of at least three independent tests for every system.

### 2.3. Characterization Techniques

#### 2.3.1. Differential Scanning Calorimetry (DSC) 

DSC scans were performed using a TA Instrument Q20 (New Castle, DE, USA) equipped with a refrigerated cooling system and nitrogen purge. Calibration was performed with indium according to the manufacturer’s recommended procedures. The uncertainty associated with each temperature is approximately ± 2 °C. About 4–6 mg of sample was sealed in an aluminium pan for every test. Thermal behavior was investigated by scanning the samples from −80 to 220 °C at a heating rate of 20 °C·min^−1^. Previous thermogravimetric analysis results show that these samples are stable until 250 °C [16]. After the first scan samples were cooled with liquid nitrogen, a second scan was immediately recorded. The midpoint of the heat capacity change was chosen to represent *T*_g_, *T*_m_ refers to the endotherm peak temperature, and the area of the endotherm peak to the melting enthalpy is Δ*H*.

#### 2.3.2. Fourier Transform Infrared-Attenuated Total Reflection Spectroscopy (FTIR-ATR) 

FTIR-ATR measurements were performed with a Thermo Nicolet Nexus FTIR spectrometer (Waltham, MA, USA) equipped with a multiple internal reflection accessory ATR single bounce. Samples were pressed against ATR accessory diamond crystal by means of the fixing screw using a flat tip. Single beam spectra of the samples were obtained after averaging 128 scans between 4000 and 400 cm^−1^ with a resolution of 4 cm^−1^. All spectra were obtained in the transmittance mode.

#### 2.3.3. Dynamic Mechanical Analysis (DMA) 

DMA was performed on a 2980 Dynamic Mechanic Analyzer (TA instruments) equipped with tensile head and reducing force option using the Custom Test and single cantilever geometry. Calibration was performed as per the manufacturer’s recommendations included in TA software, version 4.5A. The experiments were carried out on rectangular samples of dimensions close to (18.000 × 6.000 × 2.000) mm^3^. The experimental conditions employed were frequency of 1 Hz and amplitude of 30 μm (linear viscoelastic region) with a temperature ramp of 3 °C·min^−1^ and a scanning temperature range from −100 to 180 °C. These experiments yield the storage modulus (E′), the loss modulus (E′′), and the damping factor tan δ (=E′′/E′). The glass transition temperature was determined from the peak of the tan δ curve.

#### 2.3.4. Synchrotron Small-Angle X-Ray Scattering (SAXS)

SAXS experiments (samples with 10 mm of diameter and 1 mm thickness) were done on the SAXS1 beamline of the Brazilian Synchrotron Light Laboratory (LNLS). The X-ray was monitored with a photomultiplier and detected on a Pilatus (300 k, 84 mm × 107 mm) positioned at 1000 mm, generating scattering wave vectors, q, from 0.12 to 4.0 nm^−1^. The wavelength of the incident X-ray beam, λ, was 0.155 nm. Silver behenate (AgBH) was used to calibrate the diffraction angle. Polyurethane samples were placed in perpendicular position regarding the X-ray beam at room temperature. The background and parasitic scattering were determined by separate measurements on an empty holder and subtracted. 

The PU morphology can be explained through the use of a pseudo two-phase system considering that the copolymer structure is composed of periodical stacks of alternate lamellar crystals and amorphous layers [18,19]. The long period (*L*_p_), amorphous thickness (*L*_a_), and crystalline thickness (*L*_c_) were determined using Lorentz-corrected plots and the one-dimensional correlation function, γ(r) [19,20,21]. The γ(r) function was calculated according to a procedure given in the literature [18,22] with the following equation:

(1) $$\gamma (r)=\frac{\int_{0}^{\infty }I(q)q^{2}\cos(qr)dq}{\int_{0}^{\infty }q^{2}I(q)dq}=\frac{1}{Q}\int_{0}^{+\infty }q^{2}I(q)\cos(qr)dq$$

where *r* is the real space direction perpendicular to the lamellar surfaces, and *Q* is the invariant and represents the electron density difference between the hard and soft phases. In this work, the interdomain distance (*^b^L_p_*) obtained by the correlation function corresponds to the *r* value of the first maximum of the γ(r) data. The *r* value at the first zero (r_0_) is defined to be r_0_ = H(1–*V*_h_), where H is the thickness of hard domain and V_h_ corresponds to its volume fraction. The H value is determined based on the right triangle whose hypotenuse passes through at γ(r) = 1 and γ(r) = 0, and whose baseline is tangent to the γ(r) curve at its minimum [23]. By the combination of r_0_ and H data, it is possible to calculate *V*_h_ = 1–(r_0_/H). The soft domain thickness, S, is defined as the difference between H and interdomain distance: S = ^b^L_p_–H. Another important structure parameter in the PU copolymer is the average interface thickness between rigid and flexible segments, IT, which is obtained from the ratio of the hard thickness to the first minimum long period: IT = H^2^/L_pmin_ [22].

#### 2.3.5. Shore D Hardness 

Shore D hardness was measured at room temperature using a Zwick Roell (Ulm, Germany) analogical hardness testing apparatus following “UNE-EN ISO 868:1998: Plastics and ebonite. Determination of indentation hardness by means of a durometer (Shore hardness)” standard procedure at (23 ± 2) °C and 50% relative humidity.

#### 2.3.6. Tensile Properties 

Tensile properties were measured at 23 °C on five replicates of each material with an Instron Model 5582 Universal Testing machine (Grove City, PA, USA) according to “ISO 527-3 Testing method for thermoplastic polyurethane elastomers”. A 100 kN load cell was used and the cross head speed was 200 mm/min. Pneumatic grips were required to hold the test specimens.

#### 2.3.7. Durability Tests

The polyurethanes were subjected to different durability tests according to a method based on the international standard ISO 13206, ‘Thermoplastic covering films for use in agriculture and horticulture.’ These studies are as follows: (a) heating resistance: test pieces were heated in Gear oven P Selecta at 120 °C for 15, 30, and 50 days; (b) hydrolytic resistance: test pieces were immersed in water at 80 °C for 20, 40, and 60 days; (c) oil resistance: test pieces were immersed in BP Oil CS 150 at 100 °C for 10, 20, and 30 days; (d) weather resistance: test pieces were exposed in sunshine weatherometer for 200 h. Weather conditions: λ = 340 nm borosilicate filters, radiation of 35 Wm^2^ nm, T = (65 ± 3) °C; and relative humidity of 65% ± 5%. A dried cycle of 102 min was followed by 1 min of spray water (raining simulation). 

After the degradations, the material retention of tensile properties was measured following the equation:

(2) $$property\;retention\;\left(\%\right)=\;\frac{value\;after\;degradation\;test}{value\;before\;degradation\;test}\times 100$$

## 3. Results and Discussion

The current study gives valuable information about the influence of the flexible segment molecular structure and chain length on the morphology, thermal, and mechanical properties and resistance to external agents like weather, water, oil, and heat of thermoplastic polyurethanes. The main task is to evaluate the influence of the soft phase in order to tailor a polyurethane with selected properties for a specific application. So, segmented thermoplastic polyurethanes with five different flexible segment molecular structures and two molar masses (1000 and 2000 g/mol) were synthesized without solvent by the two-step method. Butanediol (BD) was used as chain extender and 4,4’-diphenylmethane diisocyanate (MDI) is the diisocyanate that was used to react with the OH groups of the polyols. The notation used in this article is PU-XY. The letter X denotes the flexible segment type, that is, X = UH, PH, UHC, PCL, or PTMG (see Table 1), and the letter Y= 100 or 200 refers to the macrodiol molar mass, that is, 1000 or 2000 g/mol. Different techniques were used to determine the degree of phase mixing rigid-flexible segments that determine polyurethanes properties, thus relating with mechanical properties and resistance to degradation under different agents.

**Figure 1:**
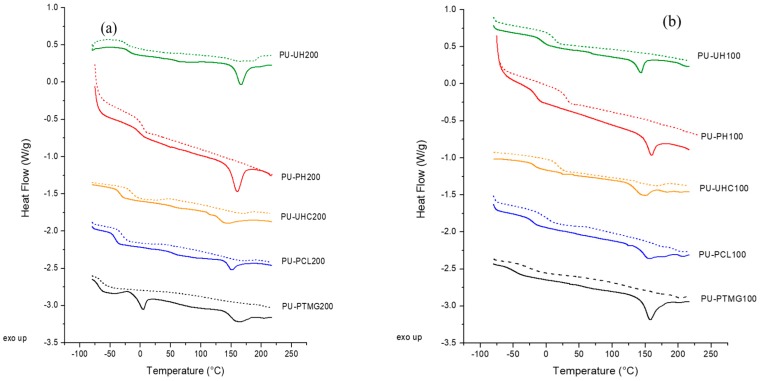
Differential scanning calorimetry (DSC) curves for the different polyurethanes (PUs) studied showing the transitions characteristics of first (continuous line) and second scans (dashed line). (**a**) system with macrodiol of 1000 g/mol molar mass and (**b**) system with macrodiol of 2000 g/mol. UH, poly(hexamethylene) carbonate diol; PH, poly(hexamethylene–pentamethylene) carbonate diol; UHC, poly(hexamethylene–caprolactone) carbonate diol; PCL, polycaprolactone polyester diol; PTMG, poly(tetramethylene ether) glycol; MDI, 4,4’-diphenylmethane diisocyanate; BD, 1,4-butanediol.

Differential scanning calorimetry (DSC) curves were obtained from the as-casted systems to determine the behavior under heat flow. Figure 1 depicts the first and the second scans of all the samples assayed as a function of temperature. All the systems show two temperature regions, with the one at a low temperature showing a glass transition temperature and the one at a high temperature as an endothermic peak [24,25,26,27]. The glass transition temperature observed at low temperature values is related to the amorphous part of the flexible segment. Values of *T*_g_ of the polyurethane strongly depend on the type of macrodiol employed in the synthesis. This value is indicative of the soft and rigid segment mixing degree. The higher the difference between the pure macrodiol or flexible segment, *T*_g,s_ (see Table 1), and the *T*_g_ of the final polyurethane, the higher miscibility or compatibility degree rigid-flexible segments [10]. In order to determine the degree of mixing macrodiol or flexible segment with rigid segment, values of the difference between the glass transition temperature of the polyurethane (*T*_g_ in Table 2) and the glass transition temperature of the macrodiol (*T*_g,s_ in Table 1), (*T*_g_ – *T*_g,s_), are compiled in Table 2. These values decrease in the following order: PH > UH > UHC > PCL > PTMG. That is, polyurethanes based on macrodiols with carbonate groups show higher *T*_g_ and *T*_g_ increment than when an ester and a more extended ether group are included in the structure. The highest phase mixing is found with PH macrodiol with odd and even carbonate groups repeating units, which increases interactions and phase mixing. UH-based polyurethanes with a homogeneous structure with six methylene repeating units decrease urethane to carbonate geometric fit and phase mixing. PU-UHC with ester linkages in addition to carbonate ones impedes geometrical fit with urethane groups, thus decreasing interactions and, consequently, phase mixing. Thus, polycarbonatediol polyurethanes depict the highest *T*_g_ values, that is, the highest flexible segment-rigid segment interaction and phase mixing. Also, the *T*_g_ increments are higher for the PCD-based PUs caused by an increase in partial mixing owing to an increase in molecular interactions, thus reflecting the best miscibility of flexible segment phase with the amorphous phase of the rigid segments [27,28]. The ester group imparts similar phase mixing characteristics. The opposite situation occurs for PTMG as macrodiol with an ether functionality that impairs molecular interaction, thus decreasing phase mixing and lowering *T*_g_. This trend is supported by looking on the molar attraction constants for ether, ester, and carbonate groups that are 256, 512, and 767 J^3/2^cm^3/2^mol^−1^, respectively. [29] Higher molar attraction and dipolar moment values of carbonate groups result, in general, in higher overall phase mixing.

At higher temperatures, 140–170 °C, there is an endotherm peak corresponding to the melting of the rigid segment phase. The value of the endotherm shifts to higher temperatures and the value of the associated enthalpy increases with the decrease in molar mass of macrodiol from 2000 to 1000 g/mol. This can be related to the formation of longer microdomains of rigid segment or structures with a greater degree of organization. This trend agrees with the results obtained in the literature [7,8,15,30,31,32].

In the second DSC scan (Figure 1), the glass transition temperature of all systems increases to higher values, indicating that the heating process increases miscibility between domains. Values of *T*_g_ and, consequently, (*T*_g_–*T*_g,s_) increase in the second scan, causing phase mixing. Neither crystallization exotherms nor melting endotherms are observed. The chains are not allowed to crystallize under the cooling process applied to the samples. Between the first and second scan, rapid cooling is carried out after reaching the melting point of the materials. This causes a rearrangement of the chains and facilitates a greater interaction between chains, increasing the miscibility between rigid and flexible segment, as can be seen in the increases in *T*_g_ for all materials. This cooling also causes the disappearance of the melting point in some of the materials owing to the short time that cooling lasts, which makes material recrystallization impossible.

Polyurethanes with macrodiol of lower molar mass (1000 g/mol) show higher values of T_g_ and (*T*_g_ – *T*_g,s_) than PUs with 2000 macrodiol molar mass (Figure 1, Table 2). This implies higher miscibility or rigid-flexible segments’ phase mixing. Furthermore, there is a gradual broadening of the glass transition with decreasing flexible segment molar mass, which is associated with increased heterogeneity in the flexible segment microdomains, as observed by other authors [29]. Increasing the molar mass from 1000 to 2000 is reflected in a decrease of the polyurethane *T*_g_, an increase in the melting temperature, and a decrease in the enthalpy (see Table 2). Lower flexible segment molar mass implies better geometrical fit owing to lower steric hindrance with the urethane groups of the rigid segments, a stronger interaction, and thus better flexible segment than with higher flexible segment molar mass because the stoichiometric relation (1:2:3) is kept constant.

The fraction of rigid segment present in the soft phase, w_H_, can be determined from values of PU’s *T*_g_. Thus, keeping in mind that the change in a thermal property of a single phase two-component system is the linear weight addition of the two individual component changes in that property, the following relation holds [33]:

(3) $$T_{g}=\left(1-w_{H,mix}\right)T_{g,s}+w_{H,mix}T_{g,H}$$

where *T*_g,s_ is the *T*_g_ of the soft phase and *T*_g,H_ is the *T*_g_ of the hard phase formed by MDI + BD, and *w*_H,mix_ is the corresponding weight fraction of rigid segment in the amorphous soft phase according to the mixing rule. The value of *T*_g,H_ = 110 °C was determined in this article, in good agreement with the value from the literature [33].

Values of *w*_H_ are depicted in Table 2 for all systems according to the first and second scan. These values decrease in the following order: UH > PH > UHC > PCL > PTMG, with the increase in molar mass of the flexible segment from 1000 to 2000. This trend is in good agreement with that observed by the *T*_g_ trends.

**Figure 2:**
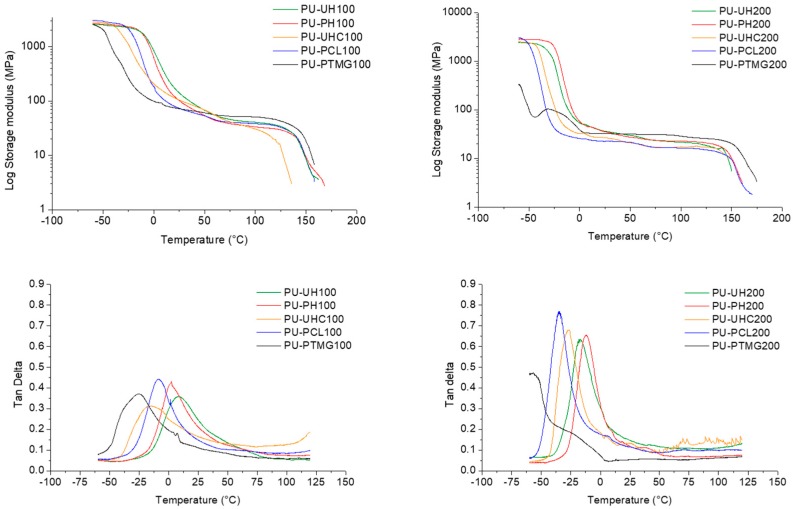
Storage modulus (E’) and tan δ of materials based on polyols with molecular mass equal to 1000 (part a) and 2000 (part b) g/mol.

Dynamic mechanical analysis (DMA) was used to obtain information on viscoelastic properties, which can be related to soft and hard microdomain thermal transitions [9,26,34,35,36]. Figure 2 shows plots of the storage modulus (E′) and the dissipation factor or tan delta (tan δ) as a function of temperature for the different polyurethanes. At a low temperature, the storage modulus shows a high and constant value characteristic of its glassy state, higher for the system with macrodiol of lower molar mass. A drop in stiffness is observed accompanying the soft domain glass transition as the temperature increases, sharper for the system with the highest polyol molar mass [9,26,36,37]. The onset of E’ decay occurs at a higher temperature for the polycarbonate-based polyols, indicating a higher ability to restore the energy supplied mechanically to the system, and is related to rigidity. The existence of an elastomeric plateau after the abrupt decrease in modulus indicates the existence of physical crosslinks because of the increase in the size of macrodiol and interconnectivity with rigid segment domains acting as physical cross-linking points. Thus, a continuous microdomain structure is developed, providing significant reinforcement and elastomeric behavior. [9,36] All the samples depict an elastomeric plateau that widens with the increase in the molar mass of the polyol [16,38]. The increase of polyol molar mass or flexible chain length in polyurethanes favors its elastomeric behavior less than for the polycarbonate-based ones. 

When the molar mass or chain length of the flexible segment decreases from 2000 to 1000 g/mol, the damping peak broadens and flattens related to the degree of ordered and the freedom of motion of molecules in the soft domains, that is, an inhibiting effect on molecular motion of the amorphous region [39,40]. Tan δ describes the ratio of storage to loss modulus and is a measure of the energy dissipation of the material. The drop in storage modulus (E′) and the peak in damping factor or tanδ is because of the glass transition of the amorphous polymer in the semi-crystalline material. As shown in Figure 2, the highest values of maximum temperature of tan δ are observed for the polycarbonate-based polyurethanes, similarly to DSC measurements. So, the trend of DMA data is consistent with that of DSC data and suggest that the miscibility between the flexible and rigid segments follows the following order: PH > UH > UHC > PCL > PTMG. This trend is the result of the greater flexibility of the etheric bond and higher phase separation in comparison with esteric and carbonate bonds. The PU-PTMG and PU-PCL systems, without polycarbonate groups, depict a higher phase segregation, and thus a higher elastomeric plateau [26,41].

**Figure 3:**
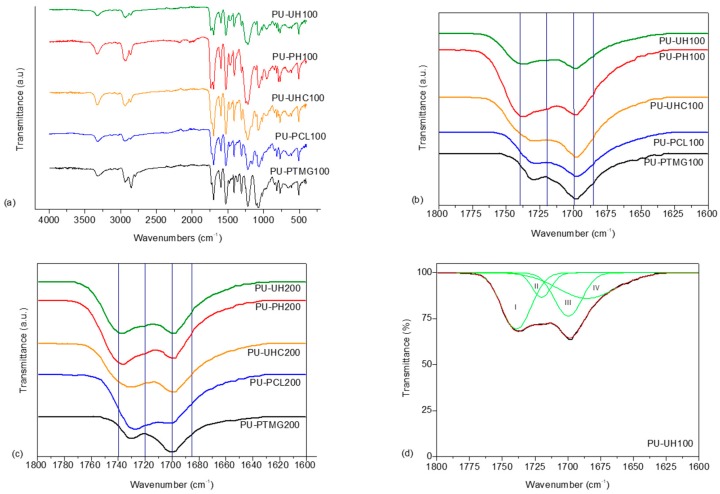
Fourier transform infrared-attenuated total reflection spectra showing transmittance spectra of (**a**) the different polyurethanes; (**b**) the carbonyl region for polyurethanes with macrodiol of 1000 g/mol; (**c**) the carbonyl region for polyurethanes with macrodiol of 2000 g/mol; and (**d**) fitting with four absorption peaks to the experimental carbonyl band of PU-UH100.

Infrared spectroscopy was used to study morphology aspects in TPUs. Figure 3a shows the FTIR spectra of the systems with macrodiol 1000 g/mol molar mass, as an example. Different absorption peaks can be used to characterize these materials, that is, the OH absorption at 3500 cm^−1^, the NH stretching vibration at 3500–3000 cm^−1^, and the absorption of NCO groups at 2260 cm^−1^ and CO group of urethane at 1800–1640 cm^−1^. A small broad peak at 3276 cm^−1^ appears, resulting from the formation of NH of the urethane linkage, while the –OH band at 3470 cm^−1^ of the corresponding polyols disappears. Simultaneously, the disappearance of the NCO stretching band at 2273 cm^−1^ as a consequence of the reaction between OH and NCO groups is observed. These facts reveal the formation of the PU consistent with the reaction of –OH with –NCO [16].

The carbonyl absorption region (1630–1730 cm^−1^) is of particular interest because it gives information about inter urethane hydrogen bonding (see Figure 3b,c). Frequency shifts to lower values occur as a result of hydrogen bonding [42,43,44,45]. Two broad bands are observed for every system that can be related to different carbonyl interactions. Thus, this absorption region can be split into four contributions for polycarbonate- and polyester-based TPUs: (1) band I at 1740 cm^−1^ related to free carbonyls of carbonates and esters, (2) band II at 1720 cm^−1^ related to associated carbonates and esters, (3) band III at 1700 cm^−1^ related to free urethanes, and (4) band IV at 1685 cm^−1^ related to associated urethanes molecular groups. For polyether-based TPUs, the bands are somewhat different; only two bands can be considered: (1) band III at 1730 cm^−1^ that corresponds to free carbonyl groups and (2) band IV at 1700 cm^−1^ that is related to H-bonded carbonyl groups [26,46].

**Scheme 1:**
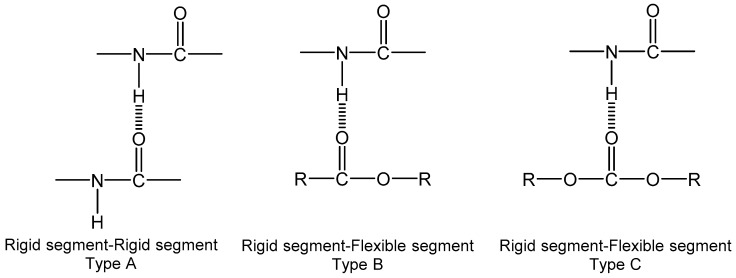
Types of associations through hydrogen bonds that may exist in polyurethane samples.

Scheme 1 shows the different types of associations through hydrogen bonding between soft and hard segments that can be found in segmented polyurethanes.

An iteration procedure of damping least squares was used to separate the absorption peaks in the carbonyl region corresponding to different kinds of hydrogen bonding with the aid of software Origin-Pro- 8 (see, as an example, Figure 3 part d).

The ratio of the area of the associated urethanes band (A_b_) (band IV) to the free urethanes ones (A_f_) (band III) can be used to calculate the fraction of hydrogen bonded urethane carbonyl groups in the rigid segment region, X_b_. By assuming an isotropic material, this absorbance ratio is related to X_b_ by the following equation [29,47]:

(4) $$\frac{A_{b}}{A_{f}}=k\frac{X_{b}}{1-X_{b}}$$

where k = 1.2 is the ratio between absorption coefficients [29,47].

The maximum rigid segment–flexible segment mixing determined by FTIR analysis, w_H,FTIR_, by assuming that H-bonded carbonyl exists only in rigid segment domains, can be calculated from the following equation [48]:

(5) $$w_{H,FTIR}=\frac{\left(1-X_{b}\right)f}{\left[\left(1-X_{b}\right)f+\left(1-f\right)\right]}$$

X_b_ and w_H,FTIR_ values of the different systems assayed are compiled in Table 2. Low values of X_b_ are indicative of a lower association of rigid segment between itself and a greater influence of flexible segment within rigid segments domains, and definitely a higher miscibility between flexible and rigid segments. The lowest Xb value is obtained for PU-PTMG, indicating a higher phase separation similar to data obtained by DSC and DMA results. These values are lower than the corresponding rigid segments values calculated from initial compositions, 0.32 for 2000 g/mol and 0.48 for 1000 g/mol.

For both System 100 and System 200, we can observe that the materials based on pure polycarbonatediols present lower X_b_ values than ester polyol polyurethane, that is, higher miscibility between both segments than the materials based on polyether, although this fact is more visible in System 200, where the content of flexible segment is higher and the segregation between segments is more favored. Values of the weight fraction of rigid segment in the soft phase calculated from FTIR analysis (Equation (5)) decrease as the molar mass of the macrodiol increases from 1000 to 2000. 

These results are in accordance with data observed previously at DSC studies, where the polyether- and polyester-based TPUs had higher tendency to segregate between rigid and flexible segments.

**Figure 4:**
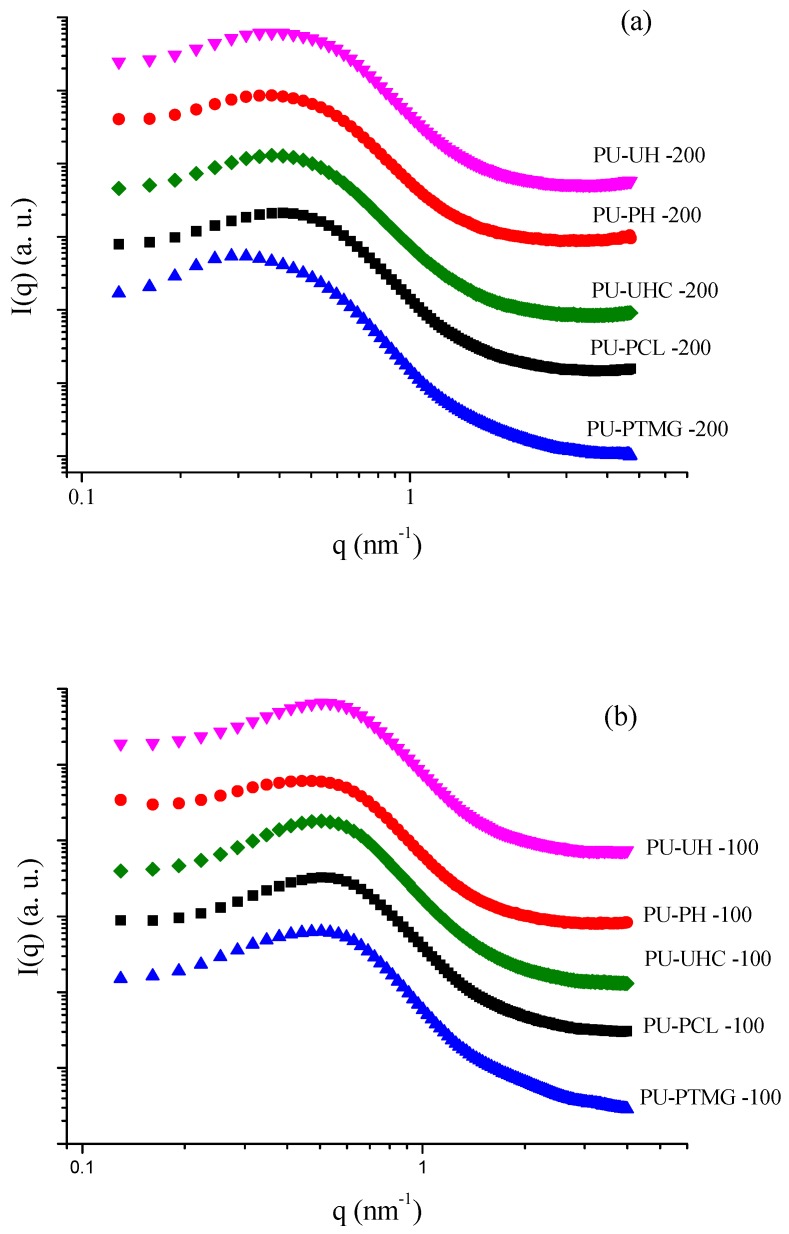
Synchrotron small-angle X-ray scattering (SAXS) patterns for polyurethanes with 1000 (part (**b**)) and 2000 (part (**a**)) polyol molar mass.

**Figure 5:**
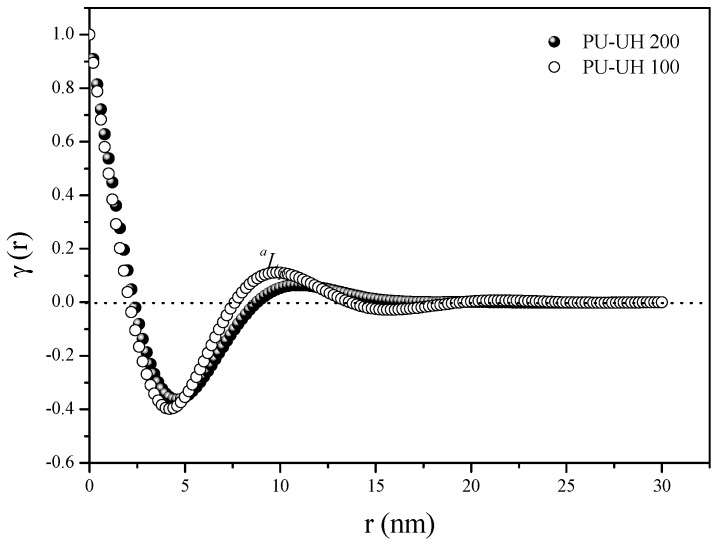
Example of correlation function for PU-UH.

Figure 4 illustrates the SAXS patterns for TPU with macrodiol of 1000 and 2000 g/mol molar mass, respectively. Desmeared data are plotted on a relative scale, that is, on the abscissa is the scattering vector, defined by q=4π/(λsin(θ)). In this work, we will adopt a mathematical procedure to determine polyurethane’s structural characteristics based on the one-dimensional correlation function, because there is a degree of continuity in its morphology [49], as the rigid segment content is between 30 wt % and 50 wt %. Table 3 summarized the SAXS constants determined by Lorentz-corrected profiles and the 1D correlation function. The TPU materials with 2000 g/mol macrodiol molar mass (32 wt % hard domain) (see Figure 4a) show a high correlation peak between 0.3 and 0.5 nm^−1^ that is characteristic of a phase-separated morphology. On the other hand, the maximum position of the q is dependent on the type of the soft block. According to the Braggs law (Lpa=2π/qmax), the size of the interdomains was between 11 and 13 nm (see Table 3). When the molecular mass of the polyol was reduced, these domains show a small reduction in values. However, the scattering intensity profile of TPUs with higher hard content shows a narrower peak (see Figure 4b), which is a result of the lower polydispersity of the blocks owing to the lower molar mass of the polyol. The ^a^L_p_ and ^b^L_p_ data show a similar trend when compared with the polyol type. The results are analogous to those found with the Teubner–Strey equation for co-continuous TPU phase morphology [49]. Figure 5 plots the correlation function for PU-UH with 1000 and 2000 g/mol macrodiol molar mass. PU-UH 100 has lower molecular weight and, as expected, the minor interdomain distance between rigid segments, as observed in Lorentz-corrected profiles ($I(q).q^{2}\;vs.\;q$). Similar results are observed in all samples when compared with the size of the polyol [49,50,51,52].

The phase separation degree of polyurethanes is influenced by the chemical interaction between blocks, hydrogen density in rigid and flexible segment, and chemical regularity of the chains [51,53]. The correlation function is an interesting tool for analyzing the effect that the constituents of the TPU have on their degree of phase separation [23,52]. The polydispersity index (*P*) of the interdomain distance can be estimated by the broadening of the SAXS curve, defined as $P=q_{0}/q_{\max}$, where q_max_ and q_0_ are the q values corresponding to the peak and intercept, respectively, on the q axis of the tangent at the inflection point. This procedure can be done more accurately using Lorentz-corrected plots [12]. The polydispersity values indicative of the interdomain distances distribution around the mean value increase with the polyol molecular weight. The larger interfacial region between the rigid and flexible blocks is a measure of the degree of interaction between the segments. Thus, the smaller interfacial region values between the blocks result in a smaller phase separation degree [49,51,52].

In this work, the hard domain thickness, H, was estimated using the one-dimensional correlation function (see Table 4). On the basis of the hypothesis that the PU morphology forms a continuous lamellar structure, the ideal hard domain thickness THS=LpaVhb can be estimated from the theoretical volume fraction of the rigid segments, Vhb, and the interdomain, Lpa, distance computed according to Leung and Koberstein assumptions [52]. The average T_HS_ value for PUs with a small flexible segment size (1000 g/mol) is ~4.7 nm and ~3.8 nm for PU with 2000g/mol. Thus, it is possible to clearly observe that the sample with PTMG presents greater separation of phases in comparison with the other PUs, once the two-phase model used approaches the theoretical value. For the carbonatediols-based PU, the specific interaction between the soft coil results in an increase of deviation of the lamellar structure, and results in a decrease of the phase separation degree and growth of the phase mixing. This hypothesis is also evident through the values of V_h_, as the greater separation of phases results in the formation of domains with greater values when compared with PUs with similar soft sizes [23,51,52].

As previously mentioned, higher values of (*T*_g_ – *T*_g,s_) result in lower phase separation and, consequently, a reduction of interface (IT) values between the blocks owing to a higher energy contribution by secondary interactions, like hydrogen and dipole bonds. A lower phase separation degree is observed in PUs with carbonate diols when compared with polyether. This behavior is independent of the molecular mass of the macrodiol and is related to the higher molar attraction and dipolar moment values of carbonate groups when compared with other polyols.

Table 4 compiles values of tensile stress at different deformations, tensile stress and elongation values at break from stress–strain plots, and hardness. The data reveal that the tensile properties of these materials are strongly related to their structural characteristics, such as rigid segment domains, intermolecular bonding, miscibility rigid, and flexible segments, as well as the possibility of the domains to crystallize under strain [46,53,54]. In general, polycarbonate-based polyurethanes (PU-UH, PU-PH, and PU-UHC) depict higher values of tensile stress and break at lower elongation values with higher tensile strength values than polyether- and polyester-based PUs. This behavior is related to the higher phase mixing rigid-flexible segment. It is also related to the highest group molar attraction constants that result in higher energy interaction of the carbonate group [27,28], developing a tighter structure with higher cohesive energy. When comparing System 200 with System 100, we observe an increase of tensile stress at different elongations and at break, as well as a decrease in strain at break. The decrease of macrodiol molar mass increases miscibility or the phase interaction between flexible and rigid segments, and the polymer becomes more rigid, making it difficult to stretch, accounting for reduced elongation. The phase segregation and hydrogen bonding decreases from System 200 to System 100 are related to a reduction of deformation at break, indicating a loss in the ability of the elastomeric material (decrease in the elastomeric behavior), thus developing an increase in tensile strength [7,8,15,16,17,52,55]. Higher rigid and flexible segment phase interaction results in higher resistance of the material to elongate owing to a higher number of hydrogen bonding interactions, more accentuated for the polycarbonate-based polyurethanes. The higher the number of urethane groups, the higher the tensile strength and the lower the elastomeric capacity. These results are in good accord with DMA data and with phase miscibility results.

Shore A hardness is higher for polyurethanes with polycarbonate diol based segment and with the lowest polyol molar mass. The increase in hardness with the decrease in the polyol molar mass is related to the formation of a more developed rigid-flexible segment interaction that builds such a kind of microdomain, providing crossover points in the material, thus reinforcing the soft matrix. This results from a higher degree of miscibility or phase mixing, as observed by DSC, FTIR, DMA, and SAXS experiments. Polyurethanes with polycarbonatediol-based macrodiols show somewhat higher hardness values owing to the carbonate higher interaction energy compared with the ester and ether groups [27,28,29].

**Figure 6:**
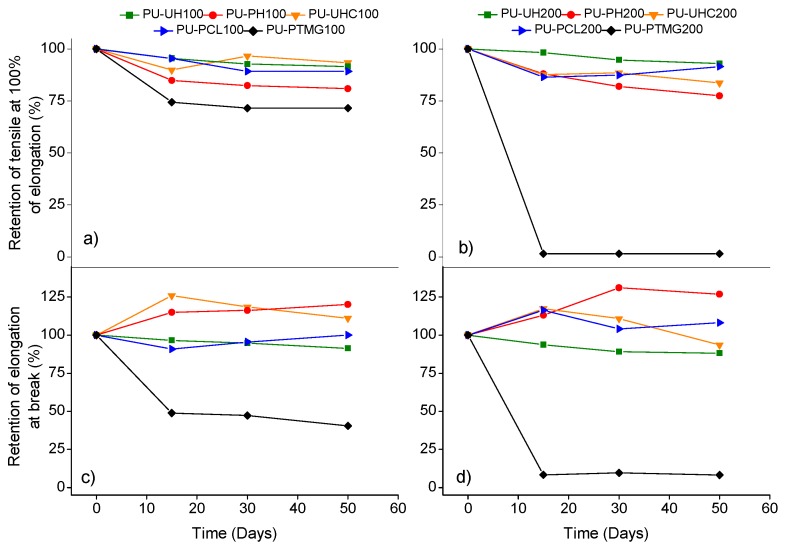
Durability test under heat resistance: (**a**) and (**b**), retention of tensile strength at 100% of elongation; (**c**) and (**d**), retention of elongation at break.

**Figure 7:**
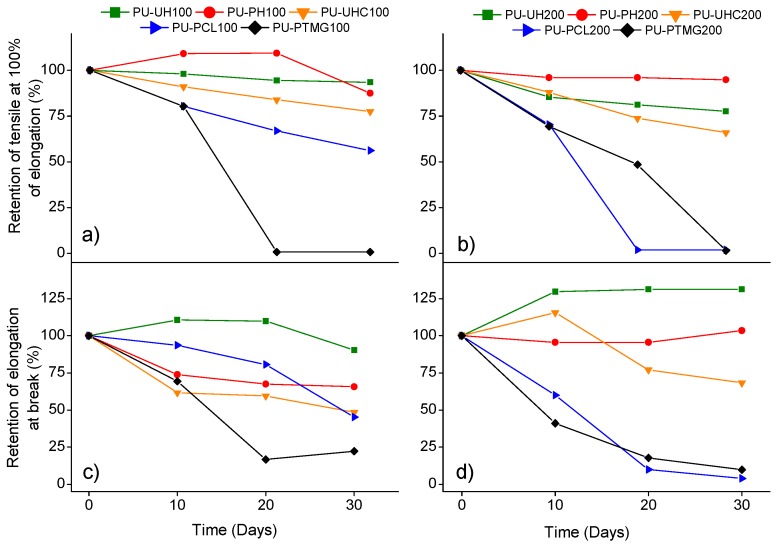
Durability test under oil resistance: (**a**) and (**b**), retention of tensile strength at 100% of elongation; (**c**) and (**d**), retention of elongation at break.

**Figure 8:**
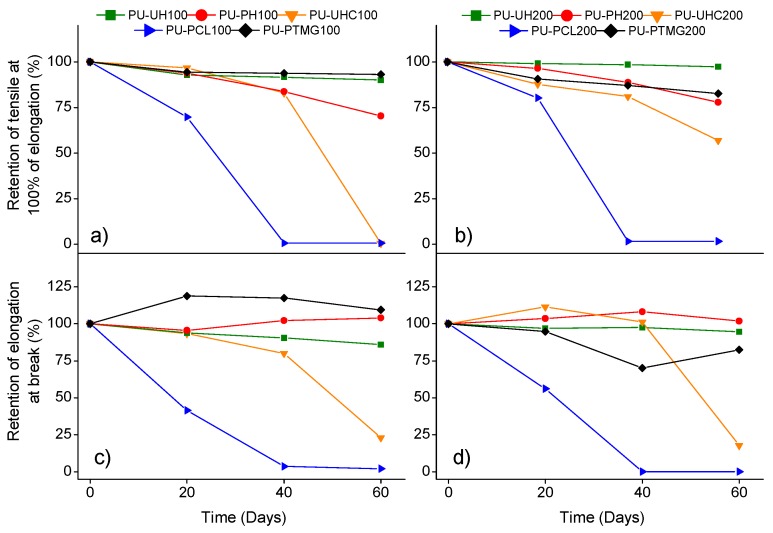
Durability test under water resistance: (**a**) and (**b**), retention of tensile strength at 100% of elongation; (**c**) and (**d**), retention of elongation at break.

**Figure 9:**
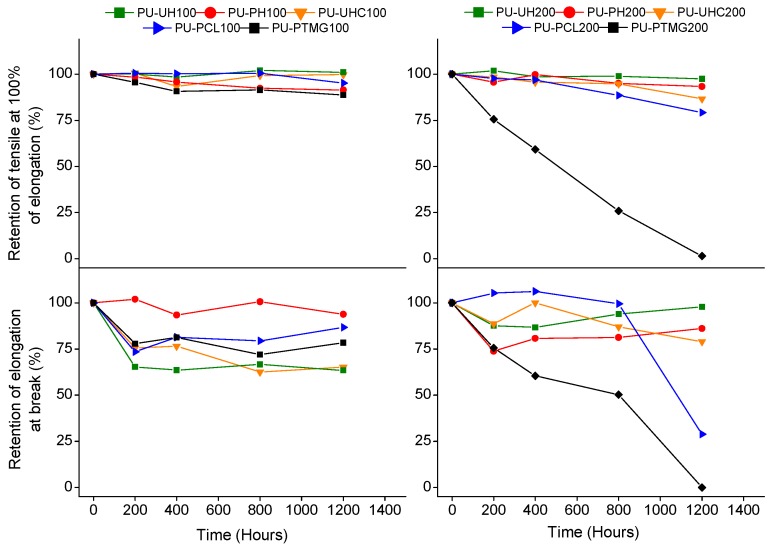
Durability test under weather resistance: (**a**) and (**b**), retention of tensile strength at 100% of elongation; (**c**) and (**d**), retention of elongation at break.

Durability test were conducted to determine the heating, hydrolytic, oil, and weather resistance of the polyurethanes under study. Reduction in tensile strength and elongation of the polyurethane after being subjected to the corresponding perturbation (see Equation (2)) was the criterion selected to assess durability [56]. Figure 6, Figure 7, Figure 8 and Figure 9 plot the retention of tensile strength at 100% elongation and retention of elongation at break for the two systems studied after subjecting the samples to heating, oil, water, and weather resistance tests, respectively. 

The first fact that should be highlighted is the excellent retention of TPUs based only on carbonatediol as flexible segment; both PU-UH and PU-PH maintain retention percentages greater than 70% in all degradation processes [57]. Because all the degradation processes are mainly based on the destabilization of intermolecular forces between the chains formed, it is to be understood that materials based on carbonate groups, given that the intermolecular forces they experience are greater, generally present better retention of the values of properties.

It is worth noting the behavior experienced by polyurethanes based on flexible segment with ether groups in water degradation. This circumstance can be explained by knowing the nucleophilic nature of water. In this case, for PU-PTMG, unlike flexible segment-based materials with carbonate or ester groups, carbonyl groups are only present in the urethane groups formed. Thus, because the density of urethane groups formed is the same in all materials, TPUs based on ether groups will have an advantage at the time of resistance to this type of degradation, as they have less electrophilic groups where water molecules can attack [29].

Comparing each of the two systems, noticeable differences are observed for System 100. The materials present, in general, better resistance to degradation processes in comparison with System 200. This can be explained from the point of view of the density of urethane groups. Given that the reaction stoichiometry is the same, for a lower molecular weight, System 100, we will have more urethane groups formed by each chain. This fact increases the intermolecular interactions that cause better behavior regarding the resistance for the degradation processes carried out.

For System 200, the density of urethane groups is lower. Thus, the chains are more vulnerable to the degradation processes that cause a loss in terms of the initial properties of the materials. Even with this, the materials based on carbonate groups in their flexible segment do not show a remarkable loss of their properties when we use macrodiol chains with a molecular weight of 2000 g/mol. As mentioned above, the density of urethane groups will be smaller, but, increasing the molecular weight of the macrodiol, we increase the number of carbonate groups, which, as we have seen in previous sections, has an important influence in terms of the increase in the energy of the intermolecular forces, which causes a lower loss of properties than expected.

## 4. Conclusions

Segmented thermoplastic polyurethanes were synthetized with 4,4’-diphenylmethane diisocyanate (MDI) and 1,4-butanediol as rigid segment and different molecular structures as flexible segment. The selected functionalities of the flexible segment are carbonatediol, eter, and ether groups. Thus, the influence of five different macrodiols acting as flexible segment was investigated. These are as follows: poly(hexamethylene) carbonate diol, poly(hexamethylene–pentamethylene) carbonate diol, poly(hexamethylene–caprolactone) carbonate diol, poly(tetramethylene ether) glycol, and polycaprolactone polyester diol. Carbonate-based polyurethanes depict a higher glass transition temperature and tanδ than ether- and ester-based PU, and thus higher phase mixing. The glass transition temperature of polyether-based PU is lower than that of polyester-based PU owing a lower possibility of ether groups to form hydrogen bonds. So, higher phase separation is observed in ether-based PU in comparison with ester- and carbonate-based PUs. DSC, DMS, SAXS, and FTIR measurements support the higher miscibility of rigid-flexible segments in the presence of ester and carbonate groups. Higher rigid and flexible segment phase interaction results in higher resistance of the material to elongate owing to a higher number of hydrogen bonding interactions, more accentuated for the polycarbonate-based polyurethanes. Also, polyurethanes based on carbonate based macrodiols exhibit excellent retention of tensile strength and elongation after being subjected to the different degradation tests.

The carbonate diols contribute to modifications of PU microdomain morphology, leading to a decrease of the interface between rigid and flexible segments. Furthermore, the specific interaction between carbonate polyols affects the PU morphology, as well as its thermal, viscoelastic, morphological, and mechanical properties. Thus, it is possible to obtain PU with tunable properties in function of their degree of interaction between the rigid and flexible segments.

## Figures and Tables

**Table 1:** Symbol, structure, molecular weight, glass transition temperature (*T*_g_), and melting temperature (*T*_m_) of the pure materials used. UH, poly(hexamethylene) carbonate diol; PH, poly(hexamethylene–pentamethylene) carbonate diol; UHC, poly(hexamethylene–caprolactone) carbonate diol; PCL, polycaprolactone polyester diol; PTMG, poly(tetramethylene ether) glycol; MDI, 4,4’-diphenylmethane diisocyanate; BD, 1,4-butanediol.

Symbol	Structure	Molecular Weight (g·mol^−1^)	*T*_g_ (°C)	*T*_m_ (°C)
UH	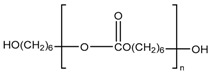	1000 2000	−69.34 −52.30	42.8 50.4
PH	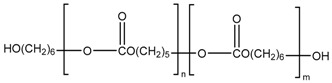	1000 2000	−56.4 −52.1	
UHC	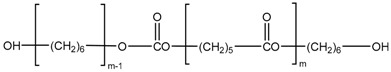	1000 2000	−66.0 −59.1	11.3 14
PCL	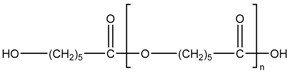	1000 2000	−71.1 −63.9	32 51.7
PTMG	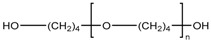	1000 2000	−75.8 −76.2	20.9 24.4
BD	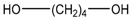	90.12	-	20.1
MDI	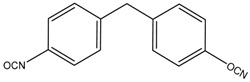	250.12	-	40

**Table 2:** Values of the glass transition temperature (*T*_g_); temperature difference (*T*_g_ – *T*_g,S_); melting temperature (*T*_m_); melting enthalpy (Δ*H*); fraction of rigid segment in the soft phase, *w*_H,DSC_; fraction of hydrogen-bonded urethane carbonyl groups in the rigid segment region, *X*_b_; and maximum rigid segment–flexible segment mixing determined by FTIR analysis, *w*_H,FTIR_. DSC, differential scanning calorimetry; FTIR, Fourier transform infrared.

System	*T*_g_ (°C)	(*T*_g_ − *T*_g,S_) (°C)	*T*_m_ (°C)	Δ*H*(J/g)	*w*_H,DSC_	*X*_b_	*W*_H,FTIR_
PU-UH100	−6.4	62.9	143.72	8.99	0.35	0.51	0.31
PU-PH100	−3.3	53.1	159.58	21.59	0.32	0.48	0.32
PU-UHC100	−17.1	48.9	148.83	17.11	0.28	0.54	0.30
PU-PCL100	−20.2	50.9	157.62	17.58	0.28	0.56	0.29
PU-PTMG100	−46.2	29.6	154.93	21.35	0.16	0.78	0.17
PU-UH200	−18.1	34.2	166.51	13.44	0.21	0.56	0.17
PU-PH200	−14.5	37.6	160.11	12.34	0.23	0.56	0.17
PU-UHC200	−30.2	28.9	148.28	10.39	0.17	0.57	0.17
PU-PCL200	−41.2	22.7	151.78	6.64	0.13	0.64	0.15
PU-PTMG200	−69.7	6.5	162.23	7.84	0.04	0.78	0.09

**Table 3:** Structural parameters obtained from synchrotron small-angle X-ray scattering (SAXS) data.

Sample	^a^L_p_ (nm)	^b^L_p_ (nm)	H (nm)	S (nm)	$V_{h}$	IT(nm)	P	T_HS_
PU-UH100	10.41	9.78	2.71	7.07	0.21	0.88	2.01	2.19
PU-PH100	10.46	10.00	2.86	7.14	0.23	0.94	1.99	2.41
PU-UHC100	10.57	10.31	2.95	7.13	0.22	0.99	1.95	2.32
PU-PCL100	10.29	9.88	3.02	6.85	0.33	1.09	1.96	3.40
PU-PTMG100	10.41	10.37	3.16	7.20	0.30	1.13	1.91	3.12
PU-UH200	11.37	10.89	2.95	7.89	0.22	0.95	2.15	2.50
PU-PH200	11.69	11.42	3.05	8.34	0.21	0.96	2.14	2.45
PU-UHC200	12.05	11.70	3.20	8.55	0.25	1.07	2.14	3.01
PU-PCL200	11.84	11.47	3.42	8.04	0.30	1.24	2.12	3.55
PU-PTMG200	13.65	13.70	3.92	9.77	0.31	1.42	2.10	4.23

^a^ Obtained by Lorentz-corrected plots; ^b^ obtained by one-dimensional correlation function.

**Table 4:** Mechanical properties of the segmented polyurethanes at 25 °C.

System	Tensile Stress at 100% (MPa)	Tensile Stress at 200% (MPa)	Tensile Stress at 300% (MPa)	Tensile Strength at Break (MPa)	Elongation at Break (%)	Hardness (Shore A)
PU-UH100	30.7 ± 0.1	52.7 ± 0.9	-	50 ± 6	210 ± 10	95.8 ± 0.2
PU-PH100	28.1 ± 0.1	49.8 ± 0.4	-	56 ± 3	235 + 16	95.0
PU-UHC100	20.4 ± 0.3	35.7 ± 0.7	-	45 ± 4	270 ± 30	94.3 ± 0.3
PU-PCL100	14.9 ± 0.2	25.8 ± 0.4	38.5 ± 0.7	46 ± 2	362 ± 7	93.0
PU-PTMG100	14.39 ± 0.02	21.8 ± 0.2	32.7 ± 0.6	47 ± 2	420 + 20	92.5 ± 0.5
PU-UH200	11.7 ± 0.5	24 ± 1	42 ± 3	49.1 ± 8.2	340 ± 20	87.3 ± 0.3
PU-PH200	11.7 ± 0.2	24 ± 0.3	40.6 ± 0.2	55 ± 2	374 ± 7	86.3 ± 0.3
PU-UHC200	8.24 ± 0.05	13.9 ± 0.2	22.9 ± 0.7	43 ± 4	480 ± 20	85.2 ± 0.2
PU-PCL200	6.107 ± 0.004	10.53 ± 0.06	17.2 ± 0.2	52 ± 4	530 + 20	82.5 ± 0.2
PU-PTMG200	6.8 ± 0.1	10.8 ± 0.2	16.4 ± 0.5	39 ± 2	600 ± 30	85.7 ± 0.3
